# Working environment of nurses in public referral hospitals of West Amhara, Ethiopia, 2021

**DOI:** 10.1186/s12912-022-00944-9

**Published:** 2022-06-24

**Authors:** Chanyalew Worku Kassahun, Addisu Taye Abate, Zewdu Baye Tezera, Debrework Tesgera Beshah, Chilot Desta Agegnehu, Mohammed Adem Getnet, Hailemichael Kindie Abate, Birhaneslasie Gebeyehu Yazew, Mahlet Temesgen Alemu

**Affiliations:** 1 Department of Medical Nursing, College of Medicine and Health Sciences, University of Gondar, Gondar, Ethiopia; 2 Department of Surgical Nursing, College of Medicine and Health Sciences, University of Gondar, Gondar, Ethiopia; 3 Community Nursing Unit, College of Medicine and Health Sciences, University of Gondar, Gondar, Ethiopia; 4 Department of Nursing, College of Health Science, Enjibara University, Injibara, Ethiopia

**Keywords:** Working environment, Nurses, University of Gondar, Ethiopia

## Abstract

**Background:**

Healthy working environment for nurses is a foundation for promoting patients’ and nurses’ safety in hospitals. However, in Ethiopia, there is scarcity of data on this issue. Therefore, the objective of this study was to assess the working environment of nurses in Public Referral Hospitals in Public Referral Hospitals of West Amhara Regional State, Ethiopia, 2021.

**Methods:**

An institution based cross-sectional study was conducted among 423 nurses from January to February 2021. Systematic random sampling was used to select nurses from each hospital. Structured, self-administered questionnaires were used to collect the data. EPI- DATA and SPSS were used for data entry and analysis respectively. Frequency, percentages, and means were calculated. Practice Environment Scale of the Nursing Work Index tool was used to measure the outcome variable. Binary and multivariable logistic regression analyses were computed to identify associated factors. Finally, texts, tables and graphs were used to report findings.

**Results:**

The response rate for the study was 96.2%. Around 210 (51.6%) of the study participants were male. One hundred eighty eight (46.2%) nurses reported that their working environment was healthy, while 219 (53.8%,) reported it as not healthy. Nurses who were working in pediatrics wards (AOR = 0.13, 0.02, 0.1) and nurses who gave care for 7–12 patients per day (AOR = 0.21, 0.05, 0.98) were less likely to have a healthy working environment, respectively. Nurses who reported the Ministry of Health to give focus to the nursing profession were 73% more likely to have a healthy work environment (AOR = 0.27; 0.09, .82).

**Conclusion:**

and recommendations.

More than half of nurses reported that their working environment was not healthy to appropriate practice. Hence, introducing systems to improve participation of nurses in hospital affairs and patient care is essential. It is also important to give attention to nurses who are working at pediatrics wards, and for nurses who give care more than the standards.

## Introduction

Nursing work environment is defined as an organizational feature that helps the nurses to engage in the work processes or limit professional nursing practice one or the other way [1–3]. The World Health Organization defines it as an environment where workers and managers collaborate to achieve sustainable protection of patients and workers way [4]. In order to realize the nurses’ potential to lead quality care and perform to the best of their abilities, they must operate in a healthy work environment that is safe, empowering, and satisfying [3]. In addition, working in a healthy environment is an important professional right for nurses that allows them to act in accordance with professional standards, legally authorized scopes of practice, and code of ethics [5]*.* The nursing working environment is too complex and characterized by: nursing involvement in hospital affairs, the basis of nursing quality, the ability, leadership and support of nurse managers, adequate staffing and resources and good professional relationships, a balanced work schedule, adequate time to meet patients’ needs and professional advancements options [1, 6, 7]. Studies reported that a positive work environment is associated with fewer occupational injuries, less burnout, and increased job satisfaction [8, 9]. It impacts the nurses’ caring behavior and loyalty to the organization [10], and resource adequacy which shows a negative effect on caring behavior and helps to improve the overall quality of nursing care [1].

Although, the nature of the work environment varies across institutional settings [11], nurses often assess their work environment as stressful and complex while meeting the physical and psychological needs of patients [8]. Nursing is inseparably linked to patient safety and poor working conditions for nurses and inadequate nurse staffing levels increase the risk for errors such as risk of health- care-associated infections and occupational injuries [12].

In a study of 12 countries in Europe showed that nurse had a concern regarding their workforce management and adequate resources, and nurses reported that important nursing tasks were often left undone because of lack of time [13]. In another study in Turkish hospital showed that, control of nursing practice’, ‘middle management accountability’ and ‘quality initiatives’ had the highest mean scores [3]. But, a single study in Ethiopia reported that the nursing environment and management was unfavorable to assure quality care [14]. In another study in Ethiopia, more 54% of the respondents had low perception to their work environment [15]. In Peru and Mexico organizational factors like resource and infrastructure deficit, work overload, job performance evaluations the working condition affect [16]. In another, cross sectional study in Shenzhen, china reported that the practice environment of nurses was satisfactory[1].

Nurses are the largest group of employees in hospitals that deliver most bedside patient care [17, 18]. It is clear that a good working environment is important in achieving patient and employee safety, and nurses can only render quality services if their work environment provides conditions that support them. However, there is limited evidence specifically on nurses’ working condition until this study. Yet, non-conducive working environments and the risks involved in these conditions cause nurses to become distracted and alienated from their profession and even leave. Considering this, undoubtedly nurses should have a positive work environment that supports superior performance and attracts them to the profession. But, before forwarding suggestions, establishing standards, and approaches, it would seem necessary to conduct a research aimed at assessing working conditions of nurses in Public Referral Hospitals of Amhara Regional State. Therefore, the objective of this study was to assess the working environment of nurses and associated factors in Public Referral Hospitals in Public Referral Hospitals of West Amhara Regional State, Ethiopia, 2021.

## Methods

### Study settings and period

This study was conducted in Public Referral Hospitals of West Amhara Regional State, Ethiopia from January to February 2021. The regional state contains; 28 million population in mid – 2018 and it has 14 Zones, three- city administrations, and 180 woredas (139 rural and 41 urban [19]. It also has 80 hospitals (8 referrals, 2 general, and 73 primaries), 847 health centers, and 3,342 health posts [20]. Despite the increased number of health facilities, shortages of skilled health personnel, medical equipment, drugs, and medical supplies, inefficient and inequitable use of health resources are the challenges of the region [21]. There are eight referral hospitals in Amhara regional state. Among them five of them (Debremarkos referral hospital, Tibebe Gion referral hospital, Felege Hiwot referral Hospital, Debre Tabor Referral Hospital and University of Gondar referral Hospital) were located in the north west part of the region where this study was conducted. Considering the resources, the University of Gondar (financial funders of this study) suggested to focus on hospitals located in the northwest part of the region. As a result, we included all the five hospitals in the study.

### Study design and population

An institution-based cross-sectional study was conducted among nurses who were working in Public Referral Hospitals in Amhara region. The source population were all nurses working in each hospital. All permanently employed nurses with work experience of equal or greater six months during and working the time of study, and who agreed to participate in the study were included.

### Sample size, sampling technique and procedures

To calculate the sample size, we considered the working condition as 50% and with an alpha error of 5%, a power of 95% and 10% of non-response rate. Then, 423 sample sizes were required for the study. Currently, there are five referral hospitals in West Amhara regional state from which samples were selected. For each hospital, the total sample size was allocated proportionally based on the number of nurses they had. Then, systematic random sampling was used to select nurses from each hospital. Then, the samples were taken from each working unit as per the sampling frame.

### Study variables

The dependent variable of the study was working environment. Age, sex, marital status, education status, position at work, professional experience,, working unit, salary, patient nurse ratio, working shift, hours worked, autonomy, flexibility schedule, participation in decision making, relationships with physicians, recognition of work, professional advancement opportunity, professional identification, satisfaction with salary were the explanatory variables.

### Operational definition

#### Nurses working environment

Composite score was computed and nursing work environment was classified as healthy if the participants scored mean and above, and not healthy if they scored below the mean [14].

### Data collection tools, measurements and procedures

The data were collected using self-administered English version questionnaires which were adapted from validated and standardized existing tools. The tools have two sections. Part-I: Socio-demographic and professional-related characteristics of nurses, and Part-II: working environment of nurses measurement scales.

The working environment was measured by the Practice Environment Scale of the Nursing Work Index [22] and which was validated in Spanish with Cronbach’s alpha coefficients of 0.90 [23]. The scale was a five-point Likert scale (5 = Strongly Agree, 4 = agree; 3 = neutral; 2 = disagree, and 1 = strongly disagree) which consisted of 32 items. Nurses indicated the degree, according to what had been presented in each item in their work. In this study the scale has an item reliability of Cronbach’s alpha coefficients of 0.92 and has five outcome subscales (nurse participation in hospital affairs -α = 0.87, nursing involvement for quality of care-α = 0.83, nurse manager ability-α = 0.8, leadership and support of nurses, staffing and resource adequacy-α = 0.76 and collegial nurse-physician relationships-α = 0.89).

The overall Practice Environment Composite score was calculated from the average of subscale scores. Then, the mean score was used to classify the working environment of nurses in to two groups (conducive and non-conducive). Respondents who scored mean (98.3 ± 18.4) and above the mean score were classified as conducive, while those who scored less than the mean score were classified as non-conducive nursing environments.

### Data management and analysis

EPI- DATA 3.1 [24] was used for data entry and SPSS version-23 software [25] for data analysis. Descriptive statistics were made using statistical measurements. Frequency, percentages, means, and standard deviations were calculated. The outcome variable was categorized as conducive and non-conducive environment. Normality tests were performed using the normal Q-Q graph and the Kolmogorov- Smirnov goodness adjustment test and Practice Environment Scale of the Nursing Work Index admit the normal model. Binary and multivariable logistic regression analyses were computed to identify associated factors. Finally, texts, tables and graphs were used to report findings.

### Quality assurance mechanisms

Before collecting the data, the face and content validity of the data collection tool was assured, checked by inviting experts in the field. The data collectors and supervisors were trained about the study purpose, and protocol. The research data collection tool was piloted (pre-tested) to check the fitness of the tool for the study settings and necessary corrections were made. The investigators exchanged all the necessary information regarding the data collection procedures with the supervisors on the daily basis. Furthermore, the respondents had been given brief information sheets to read before the filling in the questionnaires, and supervision was also done at the spot by the supervisors. In addition, detailed feedback was provided to the data collectors. The collected data were coded per operational definitions of the study variables and cheek-rechecked by the principal investigators for its completeness [26].

## Results

### Socio-demographic and professional related characteristics of nurses

Of the 423 study participants, 407 nurses responded to the questions fully that gave the response rate of 96.2%. The age of the nurses ranged from 20–65 years (mean: 31.67 ± 5.8). In terms of gender and marital status, most of the participants were male 210 (51.6%) and married 270 (66.3%) respectively. A higher proportion of the participants, 358 (88%) were degree holders. More than half of the nurses 205 (50.4%) had 5–10 years of professional experience. Around, 189(46.4%) nurses are members of professional associations. Of which 135 (33.2% were members of the Ethiopian nursing association. The majority, 316 (77.6%) of them were not satisfied with their current salary. (Table 1).

**Table 1:** Socio-demographic and professional related characteristics of nurses in Public Referral Hospitals of West Amhara Regional State, Ethiopia, 2021 (*N* = 407)

Variable category		Frequency	Percent
Age Category	< 30 years	181	44.5
	30–40 years	118	29.0
	> 40 years	108	26.5
Gender	Male	210	51.6
	Female	193	47.4
Marital Status	single	137	33.7
	ever married	270	66.3
Educational level of nurses	diploma	17	4.2
	Degree	358	88.0
	Msc and above	30	7.4
Position at work	positioned	32	7.9
	staffs	375	92.1
Professional experiences	< 5 years	133	32.7
	5–10 years	205	50.4
	> 10 years	69	17.0
Working unit category	Surgical ward	131	32.2
	Medical ward	68	16.7
	Chronic OPD	27	6.6
	OPD	30	7.4
	ICU	31	7.6
	Oby-gyn ward	10	2.5
	Emergency	24	5.9
	Pedy ward	37	9.1
	others	31	7.6
Salary category	< = 5000 birr	36	8.8
	5001–8000 birr	254	62.4
	> 8000 birr	117	28.7
Number of patient to whom the care is delivered per day	< = 6 patients	135	33.2
	7–12	105	25.8
	> 12	167	41.0
Working shift during the data collection period	morning	294	72.2
	Night	112	27.5
working hour per day	< = 8 h	192	47.2
	> 8 h	215	52.8
Are you a member of any professional association?	yes	189	46.4
	no	218	53.6
professional association that participants are members	Amhara health association	18	4.4
	Ethiopian nursing association	135	33.2
	Others^a^	4	0.9
Flexibility of your working schedule	Yes	281	69.0
	No	121	29.7
Do you have Professional identification/batch in your hospital	Yes	227	55.8
	No	177	43.5
Are you satisfied with the current salary	Yes	91	22.4
	No	316	77.6
Do you have a future vision to the nursing profession development	Yes	256	62.9
	No	146	35.9
Is there a focus of ministry of health to the nursing profession?	Yes	132	32.4
	No	273	67.1
Is there free medical services available for nurses in your hospital?	Yes	191	46.9
	No	214	52.6

^a^Amhara Public health association, Epidemiology association, Midwifery association

#### The working environment of nurses

Composite score and mean for each sub-scale and the total working environment of nurses were calculated. Accordingly, a higher mean score (33.4 ± 6.3) was observed in nursing involvement for quality of care in the hospital. More than half the participants perceived that the working environment was not conducive in terms of nurse participation in hospital affairs, 208 (51.1%) and nursing involvement for quality of care, 204 (50.1%). (Table 2).

**Table 2:** Nurses’ perception on working environment sub-scales in Public Referral Hospitals of West Amhara Regional State, Ethiopia, 2021 (*N* = 407)

Nurses’ working environment Sub-scales	Mean ± SD	Nurses’ working environment category			
		**Healthy**		**Not healthy**	
		**Frequency**	**Percent**	**Frequency**	**Percent**
Nurse Participation in Hospital Affairs	**24 ± 7.3**	**199**	**48.9**	**208**	**51.1**
Nursing involvement for Quality of Care	**33.4 ± 6.3**	**203**	**49.9**	**204**	**50.1**
Nurse Manager Ability, Leadership and Support of Nurses	**15.8 ± 4**	**245**	**60.2**	**162**	**39.8**
Staffing and Resource Adequacy	**15.8 ± 3.9**	**233**	**57.2**	**174**	**42.8**
Collegial Nurse-Physician Relationships	**9.6 ± 3**	**213**	**52.3**	**194**	**47.7**

**Fig. 1:**
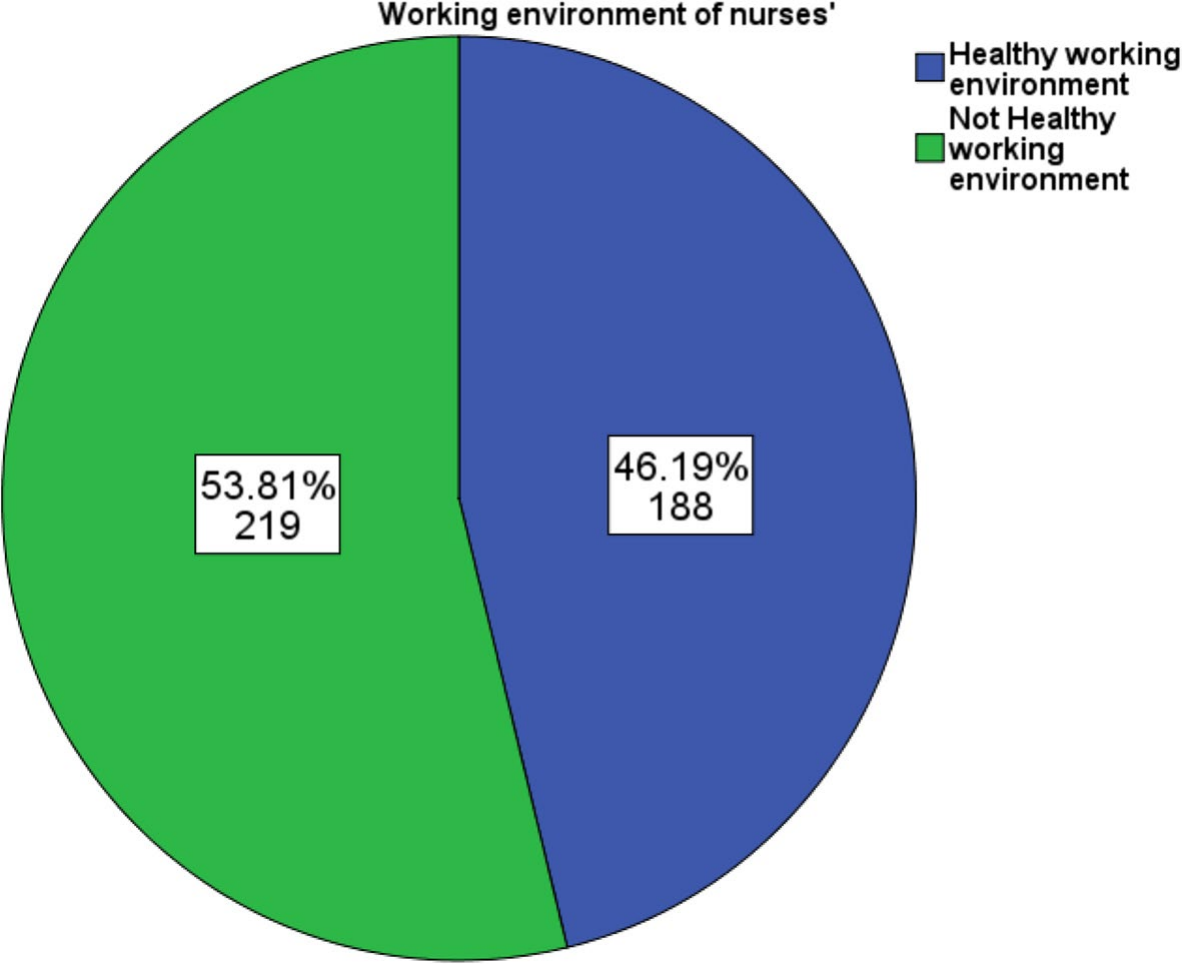
Nurses’ perception on their working environment in Public Referral Hospitals of West Amhara Regional State, Ethiopia, 2021 (*N* = 407)

The overall composite mean score for the working environment was 98.3 ± 18.4. Around 188 (46.2%, CI: 41.5%- 51.4%) perceived that their working environment was healthy, while 219 (53.8%, CI: 48.6%-58.5%) perceived it as not healthy. (Fig. 1).

#### Factors associated the nurses’ working environment

Bivariate and multivariable logistic regression analysis was carried out to see the effect of independent variables on the dependent variable. In the bivariate analysis age category from > 40 years, work experience of 5–10 years, working in chronic outpatient department, caring <  = 6 patients per day, being a members of professional association, having professional identification/batch in the hospital, being satisfied with the current salary, having a future vision to the nursing profession development, focus of ministry of health to the nursing profession were significant factors for working environment of nurse.

While working in pediatrics ward, caring 7–12 patients per day and focus of ministry of health to the nursing profession were significant factors in the multivariable logistic regression analysis.

Nurses who were working in pediatrics ward were 87% more likely to have not healthy working environment as compared to their counter parts (AOR = 0.13, 0.02, 0.1). Nurses who gave care for 7–12 patients per day had 79% less likely to have healthy environment as compared to those nursing giving care > 12 patients(AOR = 0.21, 0.05, 0.98). Nurses who perceived as ministry of health give focus to the nursing profession were 73% more likely to have healthy environment than their counter parts (AOR = 0.27, 0.09, 0.82) (Table 3).

**Table 3:** Factors associated with Nurses’ perception on working environment sub-scales in Public Referral Hospitals of West Amhara Regional State, Ethiopia, 2021 (*N* = 407)

**Variable category**		**Nurses’ working environment category**		**COR 95%CI**	**AOR 95% CI**
		**Healthy**	**Not healthy**		
Age Category	< 30 years	75	106	1	1
	30–40 years	48	70	1.03(0.64,1.65)	0.49(0.12,2)
	> 40 years	65	43	0.47(0.29,0.76)*	0.26(0.07,1.01)
Gender	Male	99	111	1	1
	Female	87	106	1.09(0.73,1.61)	1.28(0.45,3.66)
Marital Status	Single	55	82	1.45(0.95,2.2)	2.01(0.59,6.85)
	Ever married	133	137	1	
Educational level of patient	Diploma	9	8	1.02(0.32,3.35)	1.01(0.04, 26.34)
	Degree	163	195	1.37(0.65,2.89)	0.56 (0.12,2.56)
	Msc& above	16	14	1	1
Position at work	positioned	20	12	0.49(0.23,1.03)	0.39 (0.08,1.86)
	staffs	168	207	1	1
Professional experiences	< 5 years	48	85	1	
	5–10 years	112	63	0.47(0.3,0.73)*	0.29 (0.08, 1.1)
	> 10 years	28	41	0.83(0.46,1.5)	0.9,(0.16, 5.06)
Working unit category	Surgical ward	56	75	1	
	Medical ward	31	37	.89 (.494,1.607	0.77(.19, 3.14)
	Chronic OPD	18	9	.37(0.16,0.89)*	1.25(0.22,7.01)
	OPD	9	21	1.74(0.74,4.09)	0.85( .09, 7.84)
	ICU	16	15	.70 (0.32, 1.53)	2.04(0.44, 9.41)
	Oby-gyn ward	5	5	.75(0.21, 2.70)	
	Emergency	9	15	1.24 ( 0.51, 3.05)	1.82(0.2,16.68)
	Pedy ward	19	18	.71(0 .34, 1.47)	0.13(0.02,0.1)*
	others	15	16	.8( .036, 1.75)	0.35 (0.04, 3.11)
Salary category	< = 5000 birr	19	17	.74 (0.35,1.57)	1
	5001–8000 birr	116	138	.99(0.64, 1.53)	3.94(0.39, 39.6)
	> 8000 birr	53	64	1	2.01(0.64, 6.37)
Number of patient to whom the care is delivered per day	< = 6 patients	73	62	.53 (.033, .84)**	0.81(.18, 3.53)
	7–12 patients	51	54	.66(0.40,1.08)	0.21(0.05, 0.98)*
	> 12 patients	64	103	1	
Working shift during the data collection period	morning	138	156	1	
	Night	50	62	1.1(.708, 1.699)	1.07(0.38,3.02)
working hour per day	< = 8 h	83	109	1	
	> 8 h	105	110	1.25 ( 0.85,1.85)	1.58(0.41, 6.11)
Are you a member of any professional association?	yes	103	86	0.53 (0.36, 0.72)**	0.32(0.02, 6.34)
	no	85	133	1	
Flexibility of your working schedule	Yes	135	146	1	
	No	50	71	1.313(0.85,2.02)	1.26(0.40, 3.96)
Do you have Professional identification/batch in your hospital	Yes	117	110	1	
	No	70	107	1.63 (1.09, 2.421)*	1.36(0.46, 4.1)
Are you satisfied with the current salary	Yes	56	35	0.45 (0.28,.72)***	0.42(.12, 1.38)
	No	132	184	1	
Do you have a future vision to the nursing profession development	Yes	136	120	0.46(0.30, 0.70)***	1.3(0.46, 3.73)
	No	50	96	1	
Is there a focus of ministry of health to the nursing profession?	Yes	78	54	0.46( 0.30, 0.70)***	0.27(0.09, .82)*
	No	109	164	1	
Is there free medical services available for nurses in your hospital?	Yes	97	94	0.70(0.46,1.04)	1.65(0.60, 4.51)
	No	90	124	1	

* Significant, ** Highly significant, *** The significant level is <0.001

## Discussion

In this study, 46% (CI: 41.5%- 51.4%) of nurses perceived that their working environmental was healthy while around 54% (CI: 48.6%-58.5%) nurses perceived that their working environment was not healthy, especially in terms of nurse participation in hospital affairs (51.1%) and nursing involvement for quality of care (50.1%). Working in pediatrics ward, caring for 7–12 patients per day, focus of Ministry of health to the nursing profession were significant factors for working environment of nurses.

This study revealed that more than half (54%) of the nurses perceived that their working environment was not healthy. This indicates that the importance of developing nursing related work policies and procedures like nursing involvement in quality care, adequate staffing and collegial relations [27, 28]. This helps the nurse to carry out tasks efficiently to ensure that clients receive quality healthcare services [18]. The finding is higher than a study conducted in Jimma University Medical Center, Ethiopia [15]. This percentage is not consistent with the study conducted in five tertiary general hospitals in Shenzhen, China where majority of nurses reported that the practice environmental of nurses was satisfactory [1]. This difference might be due to the fact that in china new nurses’ standardization training program was introduced to improve nursing services and quality of nursing cares. But, this finding is consistent with a qualitative study finding in united kingdom where participants expressed worries over their workplace environment [29]. This finding supports the finding in a study conducted 12 countries in Europe where nurse had concerns with workforce management and adequate resources, and reported that important nursing tasks were often left undone because of lack of time [13].

Nurses who gave care for 7–12 patients per day had 79% less likely to have healthy environment as compared to those nursing giving care > 12 patients. This finding is consistent with a qualitative study conducted in Peru and Mexico where nurses reported experiencing work overload and having an excessive number of patients [16]. It also supports a study finding in Dutch where nurses stated that the number of nurses available influences how patients experience the quality of care [30].

Nurses who perceived as ministry of health give focus to the nursing profession were 87% more likely to have healthy environment than their counter parts (AOR = 0.27, 0.09, 0.82). This finding supports the ideas that leadership had an impact on the work environment of nurses [31]. It also support the fact that the use of a transformational leadership style can foster the autonomy and empowerment of nurses to cultivate a positive work environment [32].

## Limitation of the study

The interpretation of this finding should account the following limitations. The finding is based on the nurses self-report of their working environment. Hence, it would over/under report the findings. As the study is quantitative study, it might not reflect the exact perception of nurses’ view in their work environment.

## Conclusion and recommendations

More than half nurses reported that their working environment is not-healthy for appropriate practice especially in terms of nurse participation in hospital affairs and nursing involvement for quality of care. Working in pediatrics ward, caring 7–12 patients per day, focus of ministry of health to the nursing profession were significant factors for working environment of nurses.

Hence, introducing systems to improve participation of nurses in hospital affairs and patient care is essential. It is also important to give attention to nurses who are working at pediatrics ward, and for nurses who give care more that the standards.

## Data Availability

The data set generated in this study will be available upon reasonable request from the corresponding author.

## Abbreviations

AOR: Adjusted Odds Ratio
CI: Confidence interval
COR: Crude Odds Ratio
